# Books and Pamphlets Received

**Published:** 1884-06-20

**Authors:** 


### *BOOKS AND PAMPHLETS RECEIVED.*

*The Pathology*, Diagnosis and Treatment of Diseases of the Rectum and Anus, by Charles B. Kelsey, M. D., Surgeon to St. Paul's Infirmary for Diseases of the Rectum; Consulting Surgeon for Diseases of the Rectum to the Harlem Hospital and Dispensary for Women and Children, Etc., With Two Chromo-Lithographs, and Nearly One Hundred Illustrations, New York: Wm. Wood & Co., 1884.

This is a revised and enlarged edition of the work published by Dr. Kelsey in 1883. It is large and full upon the subjects treated, containing 416 large octavo pages, in which every branch of the subject is brovght up to date.

The want of information on the part of a great many practitioners in this department, and the consequent ill success of treatment is most unfortunate, if not disreputable, to those assuming to practice the Profession. Such members of the Profession, by procuring and carefully studyiug this work, will be able to find how little they do know, and will be astonished at how much more can be accomplished in this diffieult field than they have, perhaps, ever conceiued possible.

*Manual of Physiology*, a Text Book for Students of Medicine, by Gerald F. Yeo, M. D., F. R. C. S. Professor of Physiology in Kings College London, Etc. Philadelphia : P. Blakiston, Son & Co., 1012 Walnut st, 1884.

The above work is intermediate as to size, containing 749 small octavo pages. It is illustrated, neatly gotten up, and ably written by a Teacher of Physiology. Price, cloth, $4.00; leather, $5.00.

We hesitate not, to recommend this work, as in all respects an admirable Text Book for students. It is plainly and concisely written; yet sufficiently full and comprehensive upon every essential part of the science. The order and arrangement of subjects are good, and every department is brought down to the latest observations. There is a table of comparative weights and measures, and a glossary of difficult terms, with a full and satisfactory index; and last, not least, the price of the work is moderate.

*Pathology and Treatment of Gonorrhœa*, by J. L. Milton, Senior Surgeon to St. John's Hospital for the Diseases of the Skin, London. Fifth edition. New York: Wm. Wood & Co., 1884.

A work of 306 actavo pages. It is a revised and improved edition of the former excellent work of the author. It is eminently practical. The writer states that "nothing has been recommended by myself in this work but has stood the brunt, not only of experience, but of special observation. My aim was as far as possible, *to separate clearly what might be looked on as established from what was doubtful, and not merely to prove every assertion, but to place it on such a basis that it could not be disproved.*"

*Legal Medicine*, by Charles Meymott Tidy, M. B., F. C. S., Master of Surgery; Professor of Chemistry and of Forensic Medicine at the London Hospital; Officer of Health for Islingston; Late Deputy Medical Officer of Health, and Public Analyst for the City of London, Etc. New York : Wm. Wood & Co., 1884.

A book of 321 large octavo pages, being volume III of the able and interesting work on Legal Medicine, by Tidy.

The work is neatly gotten out and ably written, and treats of the following subjects : Legitimacy and Paternity; Pregnancy; Abortion; Rape; Indecent Exposure; Sodomy; Beastiality; Live Birth; Infanticide; Asphyxia; Drowning; Hanging; Strangulation; Suffocation.

The work must prove very useful to the practitioner who is called on to testtfy before the courts in respect to diffieult and important points in medico-legal investigation.

